# lncRNA polymorphism affects the prognosis of gastric cancer

**DOI:** 10.1186/s12957-022-02723-x

**Published:** 2022-08-31

**Authors:** Yanping Lyu, Shuangfeng Yang, Xuejie Lyu, Yuan-Liang Wang, Shumi Ji, Shuling Kang, Yu Jiang, Jianjun Xiang, Chenzhou He, Peixin Li, Baoying Liu, Chuancheng Wu

**Affiliations:** 1 Department of Preventive Medicine, School of Public Health, Fujian Medical University, Fuzhou, China; 2 The Key Laboratory of Environment and Health, School of Public Health, Fujian Medical University, Fuzhou, China; 3 Fuzhou Center for Disease Control and Prevention, Fuzhou, China

**Keywords:** GC, lncRNA, Gene, Polymorphism, Prognosis

## Abstract

**Background:**

Previous studies have found that lncRNA polymorphisms are associated with the prognosis of gastric cancer (GC), but the specific roles of many lncRNA polymorphism sites in gastric cancer are still unclear. Our study aims to deeply explore the relationship between genetic polymorphism of lncRNA and the prognosis of GC.

**Methods:**

The genotypes of candidate SNP locus were detected by Sequenom Mass ARRAY SNP. We deeply analyzed the association of lncRNA polymorphisms with GC prognosis by univariate and multivariate Cox regression, stratified analysis, conjoint analysis, and log-rank test.

**Results:**

We found that mutations at rs2579878 and rs10036719 loci reduced the risk of poor prognosis of GC. Stratified analysis showed that rs2795025, rs10036719, and rs12516079 polymorphisms were all associated with tumor prognosis. In addition, conjoint analyses showed that the interaction between these two polymorphic sites (rs2795025 and rs12516079) could increase the risk of poor prognosis. Multivariate analysis also found that the AG/AA genotype of rs10036719 and AG genotype of rs12516079 were independent prognostic factors. Moreover, the high expression of both *CCDC26* and *LINC02122* were shown to be associated with the poor survival status of GC patients.

**Conclusions:**

We find that the genetic polymorphism of lncRNA plays a role in the development of GC and is closely related to the survival time of patients. It could serve as a predictor of the prognosis of GC.

## Introduction

Gastric cancer (GC) is one of the fatal digestive tract tumors worldwide and is responsible for over one million new cases in 2020 and an estimated 769,000 deaths [1]. GC is a complex heterogeneous disease and is closely related to genetic alterations [2]. Increasing studies have found that single nucleotide polymorphism (SNP) is closely related to the occurrence, progression, and metastasis of GC [3–6] and is expected to become a powerful marker for its diagnosis and prognosis.

Long-chain non-coding RNA (lncRNA) has become the focus of cancer research due to its high specificity and easy detection in tissue, serum, plasma, urine, and saliva. lncRNA polymorphism can affect the outcomes of many biological processes and consequently affect the entire occurrence and development of cancer. Previous studies showed that *lncRNA HOX* transcript antisense RNA (*HOTAIR*) gene rs17720428 SNP was related with the risk and prognosis of GC in the Chinese Han population [7]. Similarly, other research indicated that specific lncRNA (*HOTTIP* and *MALAT1*) SNPs had the potential to be biomarkers in hepatocellular cancer (HCC) risk and prognosis [8]. The lncRNA growth arrest-specific 5 (*GAS5*) played an important role in the development of digestive system tumors [9], and its polymorphic site rs145204276 might induce the promoter activity of lncRNA *GAS5* to protect against the development of breast cancer [10]. However, some scholars implied that the polymorphic site rs145204276 may contribute to hepatocarcinogenesis by affecting the methylation status of the *GAS5* promoter and subsequently its transcriptional activity [11]. In addition, *lncRNA GAS5* rs145204276 can affect the prognosis of prostate cancer by regulating the expression of HMGB1 [12]. So far, there have been pieces of research involved in the association between lncRNA polymorphisms with cancer [13–15], but the specific role of lncRNA polymorphisms in GC and the corresponding mechanism is still unclear.

Our research aims to explore whether lncRNA polymorphism affects the prognosis of GC; we select 10 lncRNA polymorphism sites based on the previous results of the research group [16]. First of all, the candidate SNP genotypes are divided into four models: codominant model, dominant model, recessive model, and allele model to initially explore their relationship with the prognosis of GC. Then, we further explore its association with the prognosis of gastric cancer by stratified analysis. Besides, we explore the association between the combined effects of SNPs and the prognosis of GC. According to its results, a multivariate analysis is carried out to construct a risk model for the poor prognosis of GC. Finally, we explore the relationship between lncRNA expression and the prognosis of GC in the TCGA database. The results could serve a new avenue for the development of personalized therapy for the treatment of GC.

## Materials and methods

### Study populations and specimens

GC patient was derived from new cases in Xianyou County Hospital of Fujian Province, China. The inclusion criteria for patients are as follows: (1) the tissue sample obtained by operation or endoscopy, new cases confirmed by pathology; (2) confirmed date from April 2013 to November 2017; and (3) living in Xianyou for more than 10 years. We also applied the following exclusion criteria: (1) patients with gastric inflammation or benign lesions, (2) patients with critical conditions or inability to clearly answer questions, and (3) recurrent and relapse cases.

This study adopts a prospective case follow-up study design and obtains its complete survival information and clinical data through annual data by excerpts from all causes of death and case data and follow-up data conducted by village doctors. Finally, a total of 344 people were included in this study for follow-up analysis.

Five milliliters of fasting peripheral venous blood was collected from the patients. The blood samples were placed in EDTA anticoagulant tubes; centrifuged at 3000 g for 10 min; then packed into plasma, leukocyte, and erythrocytes; and stored in a −80 °C refrigerator. All subjects gave their consent for inclusion before they participated in the study. All procedures involving human participants were performed by the ethical standards of the institutional and national research committee and with the 1964 Helsinki Declaration and its later amendments or comparable ethical standards. The study was approved by the Bioethics Committee of the Medical University of Fujian (Fu Medical Ethics Review No. 97).

### Genotyping lncRNA-related SNPs

The genotypes of candidate SNP locus were detected by Sequenom Mass ARRAY SNP. The PCR amplification primers and single-base extension primers of the SNP site to be detected were designed using Genotyping Tools of Sequenom Company and Mass ARRAY Assay Design software. The relative molecular mass of the extension product was detected by matrix-assisted laser desorption ionization time-of-flight mass spectrometry (MALDI-TOF-MS), and the genotyping of SNP was detected by judging the differences.

### Quality control

Quality controls were carried out according to the following standards: (1) Dish QC values can be calculated from the signal values of thousands of non-polymorphic probes, which can be evaluated from the difference between the distribution of signal value and background signal value. Samples with DQC lower than 0.82 were not included in the subsequent typing; (2) a phenotype-blind genotyping during genotyping was pursued.

### Statistical analyses

The clinical staging of patients in this study was based on the latest version of the eighth edition of gastric cancer pathological staging published by AJCC. In our study, we classified patients with TNM stage I as the early stage, patients with TNM stages II–III as the middle stage, and patients with TNM stage IV as the advanced stage. The 1-, 3-, and 5-year survival rates of different genotypes at the same polymorphism site were obtained by the life table method. The median survival time (MST) of patients was obtained by the Kaplan-Meier method. Univariate and multivariate Cox regression analyses were used to analyze the relationship between lncRNA polymorphism sites and the prognosis of GC, further calculating the hazard ratios (HRs) and its 95% confidence interval (confidence intervals, CIs). Nomogram was used to visualize the results of the multivariate analysis. Log-rank test was used to explore the relationship between lncRNA expression and the prognosis of GC. A chi-square test was performed to analyze the relationship between the gene expression and clinicopathologic features in GC. The R package (DESeq) was used to analyze gene expression differences between normal and cancer tissues of GC. The SPSS 18.0 and R 4.0 software package was used to complete the above analysis. All *P* values were based on the bilateral test, and the statistical test level was *α* = 0.05.

## Results

### Screening of GC-related lncRNA SNP

A total of 10 lncRNA polymorphic loci and 344 patients were included in this study. The details on these 10 polymorphic loci are shown in Table 1, and the characteristics of the patients are presented in Table 2.

**Table 1:** Basic information of 10 candidate lincRNA SNP loci

No.	SNP ID	Gene name	Num. of transcripts	Chromosome	Cytoband	H-W ***P***	MAF
1	rs10036719	*LINC02122*	1	5	q13.3	0.834	0.358
2	rs12516079	*LINC02122*	1	5	q13.3	0.988	0.349
3	rs56093317	*LINC01137*	1	1	p34.3	0.513	0.316
4	rs61894277	*LINC02553*	1	11	q21	0.250	0.345
5	rs2795025	*LINC00687*	3	20	p12.2	0.961	0.253
6	rs11617815	*LINC00327*	3	13	q12.12	0.975	0.324
7	rs1348758	*LINC00927*	4	15	q25.1	0.874	0.385
8	rs2579878	*CCDC26*	3	8	q24.21	0.673	0.358
9	rs5829142	*LINC00298*	2	2	p25.1	0.283	0.349
10	rs9809325	*LINC00879*	5	3	q11.2	0.722	0.315

**Table 2:** Relationship between basic characteristics and prognosis of patients

Variables	***N***	MST (M)	Survival rate (%)			HR (95% CI)	***P***
			1-year	3-year	5-year		
Gender							
Male	253	29.00	72.67	44.35	37.75	1	0.953
Female	91	35.00	75.69	48.31	43.71	0.917 (0.667–1.261)	
Age (years)							
≦65	120	55.00	84.10	58.86	47.04	1	0.003*
65-	224	23.00	67.79	38.29	35.17	**1.600 (1.179**–**2.173)**	
Marriage status							
Married	320	31.00	74.30	46.81	40.83	1	0.020*
Others	24	17.00	62.50	26.17	17.45	**1.753 (1.078**–**0.851)**	
Educational level							
Primary and below	272	28.00	72.38	44.98	38.92	1	0.172
Junior high	52	28.00	76.70	38.52	33.02	1.151 (0.794–1.667)	
Senior high and above	20	73.00	80.00	69.44	62.14	0.560 (0.275–1.139)	
Occupation							
Farmers	255	28.00	71.32	41.67	36.53	1	0.107
Others	89	55.00	79.66	56.42	47.37	0.764 (0.548–1.065)	
Tumor location							
Non-cardia	179	28.00	72.07	44.68	39.76	1	0.594
Cardia	165	31.00	75.00	46.21	39.01	0.927 (0.701–1.225)	
TNM stage							
I–III	217	81.00	92.17	66.93	60.16	1	<0.001*
IV	127	10.00	41.27	8.50	4.64	**6.590 (4.889**–**8.881)**	
Operation							
No	76	8.00	35.53	3.80	1.90	1	<0.001*
Yes	268	64.00	84.27	57.17	49.92	**0.175 (0.129**–**0.238)**	
Chemotherapy							
No	140	22.00	65.00	40.48	36.49	1	0.036*
Yes	204	33.00	79.31	48.81	41.23	**0.742 (0.561**–**0.981)**	
Radiotherapy							
No	243	29.00	73.25	44.61	38.47	1	0.528
Yes	101	33.00	74.00	47.40	41.52	0.905 (0.665–1.233)	

*P < 0.05. When the MST cannot be calculated, it is replaced by the average survival time

### Health lifestyle and prognosis of patients with GC

To assess the factors affecting the survival of patients with GC, we analyzed the association between a healthy lifestyle and the survival status of GC patients. The results are shown in Table 3. Certain habits including smoking, alcohol or tea consumption, and frequent mental depression were the risk factors for a poor prognosis of GC. Self-care, sleep for more than 5 h, and regular exercise were the protective factors for the prognosis of GC (*P* < 0.05).

**Table 3:** Relationship between the health habits and lifestyle and prognosis of patients with GC after diagnosing 1 year

Variable	***N***	MST (M)	Survival rate (%)			HR (95% CI)	***P***
			1-year	3-year	5-year		
Drinking changes							
No-no	286	30.00	73.33	44.10	38.22	1	0.134
No-yes	16	20.00	75.00	31.25	25.00	**1.853 (1.035**–**3.318)**	0.038*
Yes-no	35	90.80	77.14	59.60	55.02	0.815 (0.468–1.419)	0.469
Yes-yes	7	36.00	57.14	57.14	40.82	1.362 (0.496–3.743)	0.549
Drinking tea changes							
No-no	286	30.00	74.91	45.27	38.06	1	0.003*
No-yes	39	21.00	66.67	35.60	29.67	**2.24 (1.456**–**3.447)**	<0.001*
Yes-no	27	143.12	66.67	55.33	55.33	1.08 (0.589–1.979)	0.804
Yes-yes	10	81.00	80.00	60.00	60.00	0.750 (0.301–1.865)	0.536
Drinking							
No	321	31.00	73.75	45.84	40.10	1	0.030*
Yes	23	21.00	69.57	39.13	29.92	**1.759 (1.056**–**2.93)**	
Drink tea							
No	295	31.00	74.15	46.24	39.94	1	0.008*
Yes	49	22.00	69.39	40.46	35.40	**1.701 (1.148**–**2.521)**	
Self-care							
No	62	12.00	51.61	7.60	7.60	1	<0.001*
Yes	282	41.00	78.29	53.92	46.46	**0.452 (0.322**–**0.634)**	
Depressed							
None	117	41.00	75.21	54.50	47.85	1	0.087
Seldom	131	64.00	79.31	53.23	50.03	1.078 (0.752–1.544)	0.684
Often	96	19.00	63.35	23.35	15.17	**1.458 (1.017**–**2.089)**	0.040*
Sleep time (hours/day)							
Less 5	108	17.00	60.00	30.71	24.43	1	0.001*
5–7	168	33.00	77.91	46.90	42.09	**0.681 (0.503**–**0.922)**	0.013*
More 8	68	155.71	83.82	65.09	57.99	**0.461 (0.293**–**0.724)**	0.001*
Rehabilitation exercise (times/week)							
None	168	20.00	65.37	34.88	27.97	1	0.211
1–3	102	35.00	77.45	49.67	46.66	1.042 (0.741–1.467)	0.813
3–5	36	50.00	83.10	56.92	44.15	0.940 (0.555–1.592)	0.818
>5	38	81.00	89.47	70.20	66.40	**0.548 (0.306**–**0.983)**	0.044*

*P <0.05

### lncRNA-related SNP and prognosis of patients with GC

In this study, a total of 10 lncRNA polymorphic loci were examined by univariate analysis. The results are shown in Tables 4 and 5. Interestingly, at the *CCDC26* rs2579878 locus, we found the survival rate of GC patients with the C allele was higher than that of patients with the T allele, which could also be observed in the codominance model. Similarly, at the *LINC02122* rs10036719 locus, the survival rate of patients with the A allele was higher than that with the G allele, which was also reflected in its recessive model.

**Table 4:** The relations between polymorphism site of *CCDC26* rs2579878 and prognosis of patients with GC

rs2579878	***N***	MST (M)	Survival rate (%)			HR (95% CI)	***P***
			1-year	3-year	5-year		
Codominance							
TT	141	27.00	70.92	40.59	35.25	1	0.087
TC	159	31.00	73.50	46.77	40.22	0.830 (0.612–1.124)	0.228
CC	44	136.05	81.61	57.08	49.71	**0.581 (0.354–0.954)**	0.032*
Allele gene							
T	441	28.00	71.85	42.82	37.08	1	0.030*
C	247	36.00	76.38	50.19	43.52	**0.790 (0.638–0.977)**	
Dominant model							
TT	141	27.00	70.92	40.59	35.25	1	0.080
TC+CC	203	34.00	75.25	48.82	42.22	0.771 (0.576–1.032)	
Recessive model							
TT+TC	300	29.00	72.29	43.87	37.93	1	0.066
CC	44	136.05	81.61	57.08	49.71	0.645 (0.404–1.03)	

*P was adjusted according to age, sex, TNM stage, operation, and chemotherapy

**Table 5:** The relations between polymorphism site of *LINC02122* rs10036719 and prognosis of patients with GC

rs10036719	***N***	MST (M)	Survival rate (%)			HR (95% CI)	***P***
			1-year	3-year	5-year		
Codominance							
GG	45	19.00	66.67	45.67	41.70	1	0.093
AG	155	28.00	75.41	42.03	36.89	0.851 (0.555–1.303)	0.458
AA	144	34.00	73.52	48.99	41.31	0.654 (0.422–1.012)	0.057
Allele gene							
G	245	28.00	72.19	43.27	38.54	1	0.029*
A	443	31.00	74.18	46.59	39.79	**0.796 (0.649–0.977)**	
Dominant model							
GG	45	19.00	66.67	45.67	41.70	1	0.161
AG+AA	299	30.00	74.50	45.42	39.05	0.749 (0.5–1.122)	
Recessive model							
GG+AG	200	28.00	73.43	42.78	37.88	1	0.042*
AA	144	34.00	73.52	48.99	41.31	**0.742 (0.556–0.989)**	

*P was adjusted according to age, sex, TNM stage, operation, and chemotherapy

### Stratified analysis of SNP

After combined stratification of age and TNM stage, we found 3 polymorphic loci out of 10 lncRNA-related SNPs to be associated with the prognosis of GC. From both codominant and recessive models, we found that the CC genotype at *LINC00687* rs2795025 to be a risk factor for poor prognosis in patients younger than 65 years of age with advanced GC. The AA or AG genotype at *LINC02122* rs10036719 and the GG genotype at *LINC02122* rs12516079 were protective factors for the prognosis of patients older than 65 years of age with early- or middle-stage GC (Tables 6, 7, and 8).

**Table 6:** Stratified analysis of LINC00687 polymorphism rs2795025 and prognosis of GC

rs2795025	≦65						>65					
	Stages I–III			Stage IV			Stages I–III			Stage IV		
	***N***	MST (M)	HR (95% CI)	***N***	MST (M)	HR (95% CI)	***N***	MST (M)	HR (95% CI)	***N***	MST (M)	HR (95% CI)
Codominance												
TT	46	73.67	1	19	10.00	1	81	157.29	1	56	9.00	1
TC	35	106.04	1.157 (0.525–2.554)	9	21.00	0.696 (0.282–1.717)	38	75.00	0.901 (0.475–1.709)	32	8.00	0.860 (0.538–1.374)
CC	9	73.00	1.446 (0.406–5.151)	2	4.00	5.911 **(1.103–31.671)**	8	27.00	1.936 (0.675–5.553)	9	11.00	1.087 (0.503–2.348)
Dominant model												
TT	46	73.67	1	19	10.00	1	81	157.29	1	56	9.00	1
TC+CC	44	73.00	1.209 (0.576–2.540)	11	19.00	0.872 (0.380–2.002)	46	75.00	1.029 (0.572–1.850)	41	9.00	0.901 (0.584–1.391)
Recessive model												
TT+TC	81	103.76	1	28	13.00	1	119	81.00	1	88	8.00	1
CC	9	73.00	1.364 (0.400–4.648)	2	4.00	6.611 **(1.257–34.775)**	8	27.00	1.996 (0.707–5.634)	9	11.00	1.148 (0.541–2.437)

**Table 7:** Stratified analysis of LINC02122 polymorphism rs10036719 and prognosis of GC

rs10036719	≦65						>65					
	Stages I–III			Stage IV			Stages I–III			Stage IV		
	***N***	MST (M)	HR (95% CI)	***N***	MST (M)	HR (95% CI)	***N***	MST (M)	HR (95% CI)	***N***	MST (M)	HR (95% CI)
Codominance												
GG	13	73.00	1	4	6.00	1	17	33.00	1	11	8.00	1
AG	38	105.24	1.206 (0.384–3.783)	13	10.00	0.720 (0.202–2.560)	61	81.00	0.592 (0.279–1.256)	43	9.00	0.935 (0.457–1.914)
AA	39	72.25	1.206 (0.387–3.752)	13	19.00	0.554 (0.148–2.081)	49	115.44	0.377 **(0.165**–**0.859)**	43	10.00	0.676 (0.333–1.374)
Dominant model												
GG	13	73.00	1	4	6.00	1	17	33.00	1	11	8.00	1
AG+AA	77	101.82	1.206 (0.414–3.512)	26	12.00	0.643 (0.189–2.181)	110	147.15	0.490 **(0.241**–**0.997)**	86	9.00	0.780 (0.398–1.530)
Recessive model												
GG+AG	51	96.94	1	17	10.00	1	78	75.00	1	54	8.00	1
AA	39	72.25	1.050 (0.503–2.192)	13	19.00	0.727 (0.317–1.668)	49	115.44	0.562 (0.302–1.046)	43	10.00	0.713 (0.461–1.100)

**Table 8:** Stratified analysis of LINC02122 polymorphism rs12516079 and prognosis of GC

rs12516079	≦65						>65					
	Stages I–III			Stage IV			Stages I–III			Stage IV		
	***N***	MST (M)	HR (95% CI)	***N***	MST (M)	HR (95% CI)	***N***	MST (M)	HR (95% CI)	***N***	MST (M)	HR (95% CI)
Codominance												
AA	11	75.08	1	2	4.00	1	15	75.00	1	10	8.00	1
AG	35	105.48	1.665 (0.459–6.031)	13	13.00	0.511 (0.103–2.528)	57	46.00	0.997 (0.432–2.297)	42	8.00	1.022 (0.483–2.164)
GG	44	71.86	1.420 (0.400–5.035)	15	12.00	0.522 (0.107–2.541)	55	117.32	0.535 (0.217–1.32)	45	10.00	0.729 (0.350–1.519)
Dominant model												
AA	11	75.08	1	2	4.00	1	15	75.00	1	10	8.00	1
AG+GG	79	101.38	1.522 (0.452–5.127)	28	12.00	0.517 (0.112–2.391)	112	81.00	0.767 (0.343–1.715)	87	9.00	0.837 (0.414–1.692)
Recessive model												
AA+AG	46	73.00	1	15	13.00	1	72	46.00	1	52	8.00	1
GG	44	71.86	0.965 (0.462–2.016)	15	12.00	0.941 (0.416–2.132)	55	117.32	0.536 **(0.291**–**0.987)**	45	10.00	0.717 (0.465–1.106)

### Combined effects of GC-related lncRNA polymorphism

Based on the above analysis, the TT genotype of rs2579878, the CC genotype of rs2795025, the GG genotype of rs10036719, and the AG genotype of rs12516079 led to a poor prognosis of GC. Interestingly, patients carrying both rs2795025 CC and rs12516079 AG alleles had a higher risk of poor prognosis, while other gene polymorphisms had no significant combinatory effects, which is shown in Table 9 (*P* > 0.05).

**Table 9:** The combined action of gene polymorphism loci

SNP loci	Num. of bad genotypes	***N***	MST(M)	HR (95% CI)	***P***
rs2579878*rs2795025	0	183	36.00	1	0.062
	1	98	24.00	**1.386 (1.036–1.855)**	0.028*
	2	8	36.00	1.749 (0.704–4.343)	0.228
rs2579878*rs10036719	0	178	35.00		0.087
	1	146	24.00	**1.349 (1.004–1.814)**	0.047*
	2	20	33.00	1.546 (0.854–2.798)	0.150
rs2579878*rs12516079	0	197	34.00	1	0.247
	1	84	30.00	1.206 (0.859–1.692)	0.280
	2	63	23.00	1.334 (0.926–1.921)	0.122
rs2795025*rs10036719	0	279	31.00	1	0.121
	1	57	18.00	1.393 (0.973–1.996)	0.070
	2	8	73.00	1.764 (0.645–4.824)	0.269
rs2795025*rs12516079	0	180	33.00	1	0.054
	1	153	30.00	1.227 (0.915–1.644)	0.171
	2	11	11.00	**2.122 (1.089–4.138)**	0.027*
rs10036719*rs12516079	0	160	34.00	1	0.056
	1	176	28.00	1.310 (0.981–1.748)	0.067
	2	8	9.00	2.194 (0.979–4.917)	0.056

*P <0.05

### Multifactor analysis

The 10 lncRNA polymorphic loci were included in the multivariate Cox regression analysis, as shown in Table 10. In model 1, none of the 10 polymorphic loci was associated with the prognosis of GC. After adjusting for possible confounding factors, 2 of 10 polymorphic loci were associated with the prognosis of GC patients. Among them, AG and AA genotypes of rs10036719 were shown to be protective factors for the prognosis of GC, while the AG genotype of rs12516079 was shown to associate with poor prognosis of GC patients.

**Table 10:** Cox regression survival analysis in patients with GC

SNP loci	Genotype	Model 1^a^		Model 2^b^	
		HR (95% CI)	*P*	HR (95% CI)	*P*
rs2579878	TT	1	0.253	1	0.448
	TC	0.864 (0.638–1.171)	0.346	0.910 (0.65–1.272)	0.580
	CC	0.667 (0.403–1.102)	0.114	0.712 (0.419–1.21)	0.210
rs5829142	INs	1	0.822	1	0.795
	DEl/INS	1.180 (0.677–2.054)	0.560	1.085 (0.589–1.998)	0.794
	DEL	1.194 (0.679–2.097)	0.538	0.971 (0.524–1.799)	0.927
rs11617815	AA	1	0.949	1	0.681
	GA	0.946 (0.526–1.702)	0.853	0.858 (0.458–1.606)	0.631
	GG	0.921 (0.51–1.664)	0.785	0.778 (0.401–1.508)	0.457
rs1348758	GG	1	0.851	1	0.485
	TG	0.975 (0.624–1.523)	0.911	0.873 (0.542–1.404)	0.574
	TT	0.898 (0.561–1.437)	0.653	0.754 (0.461–1.232)	0.260
rs2795025	TT	1	0.453	1	0.314
	TC	0.917 (0.668–1.259)	0.593	0.975 (0.701–1.357)	0.882
	CC	1.304 (0.776–2.193)	0.316	1.484 (0.872–2.525)	0.145
rs9809325	AA	1	0.925	1	0.227
	AG	0.976 (0.725–1.314)	0.874	1.065 (0.785–1.446)	0.686
	GG	0.894 (0.508–1.572)	0.697	1.684 (0.930–3.049)	0.085
rs10036719	GG	1	0.210	1	0.017*
	AG	0.503 (0.235–1.078)	0.077	**0.282 (0.116–0.680)**	0.005*
	AA	0.520 (0.184–1.468)	0.217	**0.226 (0.067–0.767)**	0.017*
rs61894277	TT	1	0.330	1	0.809
	TC	1.251 (0.923–1.696)	0.150	0.902 (0.650–1.251)	0.536
	CC	1.029 (0.603–1.755)	0.917	0.893 (0.512–1.557)	0.690
rs56093317	GG	1	0.471	1	0.086
	AG	1.167 (0.86–1.585)	0.321	1.197 (0.864–1.660)	0.280
	AA	0.895 (0.524–1.528)	0.683	0.628 (0.355–1.112)	0.110
rs12516079	AA	1	0.137	1	0.075
	AG	2.330 (0.991–5.478)	0.052	**2.999 (1.155–7.785)**	0.024*
	GG	1.943 (0.643–5.876)	0.239	3.541 (0.955–13.126)	0.059

^a^Model 1 does not adjust

^b^Model 2 was adjusted according to the basic characteristics and the health habits and lifestyle after the illness

*P <0.05

### Nomogram prediction model

**Fig. 1:**
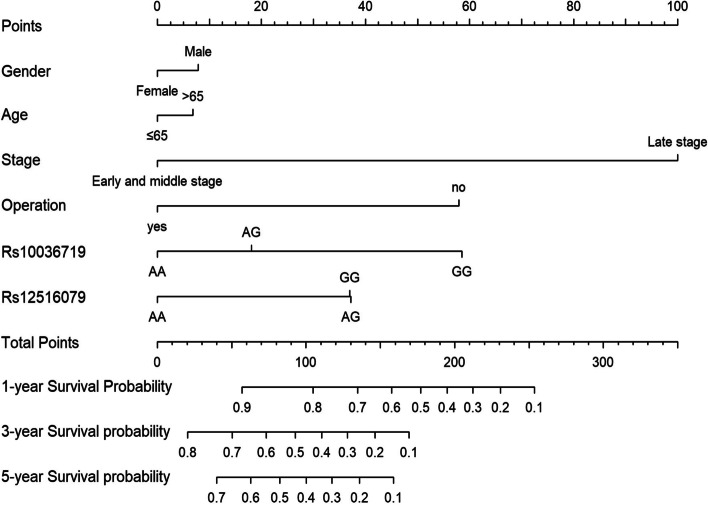
Nomogram. Gender, age, TNM staging, surgery, chemotherapy, and two polymorphic sites were included in the nomogram model

**Fig. 2:**
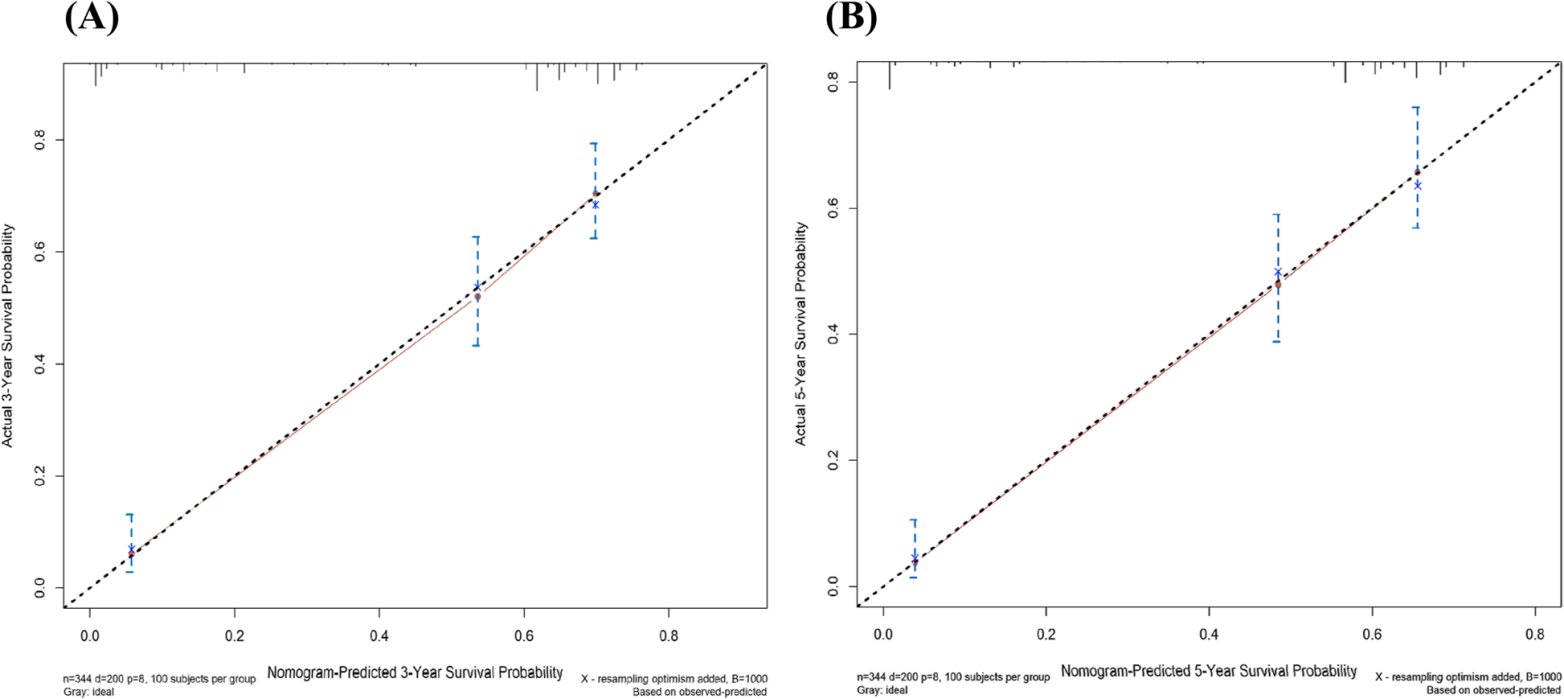
Calibration curve for **A** 3-year and **B** 5-year survival probability. The *X*-axis means nomogram-predicted survival probability, and the *Y*-axis means actual survival probability

We incorporated the polymorphic sites with statistical significance from above multivariate analysis to establish a nomogram and further evaluate their prediction performance. As shown in Fig. 1, TNM staging accounted for the largest proportion in the chart and had the greatest impact on the prognosis. The C index of the whole nomogram is 0.762, indicating that the predictive ability of the model was moderate, as shown in the calibration curve (Fig. 2).

### The relationship between the expression of the lncRNA and the prognosis of GC

**Fig. 3:**
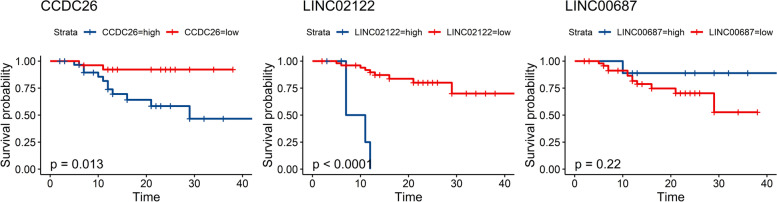
K-M survival curve plots

In the above studies, we found that four polymorphism loci of three lncRNA were associated with the survival outcome of GC patients. SNPs often affected disease by affecting gene expression; thus, we downloaded the RNA-seq data of Asian gastric adenocarcinoma patients from the TCGA database to further explore the relationship between gene expression and GC prognosis. The results showed that the high expression of both *CCDC26* and *LINC02122* were shown to be associated with the poor survival status of GC patients (Fig. 3). The cutoff value of the expression of CCDC26, LINC02122, and LINC00687 were 0.0078, 0.1763, and 0.0784, respectively, and the median values of these three genes’ expression were 0.0133, 0, and 0.0103, respectively. In addition, we also found that gender was associated with the expression of CCDC26 (*P* = 0.005), while other clinicopathologic features did not show a correlation with the expression of these genes. Unfortunately, there was no significant difference in the expression of the three lncRNAs in GC tissues and normal tissues.

## Discussion

Numerous studies have shown that long non-coding RNAs (lncRNAs) behave as a potential carcinogenic role during multiple cancer processes, such as cell proliferation, apoptosis, migration, and invasion [17–20]. It can also affect the prognosis of cancer by acting on key signaling pathways and altering the invasiveness of cancer cells [21–24]. In this study, we deeply explored the relationship between lncRNA polymorphisms and GC prognosis. Through univariate Cox analysis and stratified analysis, we found four lncRNA polymorphism loci associated with gastric cancer prognosis. Afterward, we further explored their combined effects through conjoint analysis and evaluated their predictive performance through the multivariate analysis. Finally, we explored the association between lncRNA expression and gastric cancer prognosis in the TCGA database.

In this study, we found that polymorphism of *CCDC26* was associated with the prognosis of GC. *CCDC26*, or coiled-coil domain-containing 26, is a long non-coding RNA located on the 8q24 chromosome. Previous studies have shown that lncRNA *CCDC26* levels were correlated with tumor size, tumor number, and reduced overall survival in pancreatic cancer [25]. *CCDC26* participates in cancer cell growth and apoptosis by regulating the expression of *PCNA* and *Bcl2* [25]. *CCDC26* promotes thyroid cancer malignant progression via miR-422a/*EZH2*/*Sirt6* axis [26]. Silencing of *CCDC26* can strongly reduce the wound closing rate and the number of invasive cells and further regulates the growth and metastasis of gliomas [27]. *CCDC26* can affect the drug sensitivity in gastrointestinal stromal tumors and the prognosis [28]. Besides, scholars also found the polymorphism of *CCDC26* related to cancer risk [29–31]. However, the correlation between the polymorphism of *CCDC26* and GC prognosis had not been found yet. Our research found that the patients with C mutation at the *CCDC26* rs2579878 locus had a higher survival probability and the expression of *CCDC26* could affect the survival of patients. The *CCDC26* polymorphism in GC may inhibit its expression, then reduce the number of invasive cells, and improve the prognosis. However, mechanisms underlying *CCDC26* polymorphism and its clinical significance in GC remained to be further investigated.

*LINC02122* is located on chromosome 5q13.3. The gene *IQGAP2* located on the same site was reported to be a tumor suppressor gene for prostate cancer [32]. Previous studies found 5q13.3 deletions in myeloid tumors [33]. Other studies have also indicated that loss of heterozygosity in 5q13.3 was related to the progression and metastasis of colon cancer [34]. Genome-wide DNA copy number analysis implied that focal recurrent genomic losses were observed in chromosome regions 5q13.3 of desmoplastic infantile ganglioglioma (DIG) and desmoplastic infantile astrocytoma (DIA) [35]. Furthermore, loss of copy number at 5q13.3-q35.3 is correlated with a higher histological grade of urothelial carcinomas (UCs) [36]. In our study, we found *LINC02122* polymorphisms sites rs10036719 and rs12516079 were associated with the prognosis of GC and the expression of *LINC02122* also played a role in patient survival. In addition, *LINC02122* rs10036719 A and rs12516079 G mutant alleles were beneficial to the survival of GC patients. The multivariate analysis also showed the *LINC02122* rs10036719 A mutant allele to be an independent factor for the prognosis of GC. However, there is no report about the relationship between the polymorphism of chromosome 5q13.3 and GC. Our research is the first time to reveal the relationship between them. More research is needed to further explore its role and mode of action in GC in the future.

We found polymorphic site rs2795025 of *LINC00687* was a risk factor for the poor prognosis of GC. A study based on weighted gene co-expression network analysis (WGCNA) and the linear models for microarray data analysis (LIMMA) found that *LINC00687* could be one of the important hub nodes involved in the pathogenesis of periodontitis [37]. *LINC00687* is located in 20p12.2. Genome-wide analysis of genetic variants suggested that this locus could influence the effectiveness of platinum-based chemotherapy for small-cell lung cancer (SCLC) [38]. Although the relationship between *LINC00687* polymorphism and GC has not been found, our research has shown that it is involved in the development process of GC, which was conducive to further research on the complex regulatory mechanism of GC.

Our study found that the expression of these three lncRNAs was correlated with the prognosis of GC, but there was no significant correlation with its occurrence. Previous studies have not found that the expression of these genes is related to the occurrence of GC. It is possible that some genes act in different stages, and these genes mainly affect the prognosis stage of tumors. In addition, the tumor microenvironment is extremely complex and in a constantly changing process, and the roles of gene expression in it are also complex and diverse. Some scholars [39] have also discovered the contradictory phenomenon that genes which are highly expressed in tumors have better prognosis. The specific mechanism of gene expression in the tumor microenvironment needs to be further explored.

Consistent with previous research, we found that age, TNM stage, operation, and chemotherapy were significantly related to the prognosis of GC [40–42]. In addition, changes in drinking habits were adverse factors in the prognosis of GC. Other factors that were associated with the survival time of GC patients include self-care, depression, sleep duration, and exercise.

Although the findings of our study can provide clues for the study of GC mechanism and the exploration of regulatory networks, there were ethnic and regional differences in gene polymorphisms, so the generalization of the conclusions of this study needed to be considered. Since different detection techniques and methods in the genetic testing process may also cause different detection results and the sample size of our research was still small, the conclusion needed to be further verified in a large-sample multi-center study.

## Conclusions

In conclusion, we found four lncRNA-related polymorphisms were closely related to the prognosis of GC by multivariate and stratified analyses. In addition, the interaction between polymorphous loci rs2795025 and rs12516079 could increase the risk of poor prognosis of GC. To further visualize the results of the multivariate analysis, we included gender, age, TNM staging, surgery, chemotherapy, and statistically significant polymorphic sites rs10036719 and rs12516079 in the multivariate analysis to draw a nomogram. According to the nomogram, we calculated the total score according to various indexes of patients with GC and then speculate their 1-, 3-, and 5-year survival rates. The nomogram map proved to be able to successfully predict the prognosis of patients with GC and therefore could become one of the prognostic markers for future clinical studies. Our study was helpful to understand the development trend of the GC, predict the prognosis of patients, help clinicians make corresponding treatment decisions, ultimately achieve the purpose of prolonging the life of patients, and improve the life quality of patients. At present, molecular epidemiological research was still the focus of current research. We expected that with the continuous expansion and deepening of research, the prognostic factors of GC would continue to be clarified and make individualized treatment possible. Due to the differences in race, region, technology, and detection methods, it was desirable to verify our current results with a larger population.

## Data Availability

The datasets used and/or analyzed during the current study are available from the corresponding author on reasonable request.

## Abbreviations

GC: Gastric cancer
SNP: Single nucleotide polymorphism
lncRNA: Long-chain non-coding RNA
MALDI-TOF-MS: Matrix-assisted laser desorption ionization time-of-flight mass spectrometry
H-W: Hardy-Weinberg genetic equilibrium
CCDC26: Coiled-coil domain-containing 26
